# Clinical efficacy of Nafamostat Mesylate in combination with Favipiravir for COVID-19 pneumonia treatment review article

**DOI:** 10.1016/j.amsu.2021.102560

**Published:** 2021-07-14

**Authors:** Maram H. Abduljabbar

**Affiliations:** Department of Pharmacology and Toxicology, College of Pharmacy, Taif University, Taif, 21944, Saudi Arabia

**Keywords:** COVID-19, Nafamostat mesylate, Favipiravir, Pneumonia, SARS-CoV

## Abstract

Alleviation and treatment of the extensive detrimental implications of COVID-19 have materialised into the primary objectives of scientists and virologists along with pathologists assigned with the responsibility of provisioning of care for infected patients. Development and introduction of vaccines have been, till to date, primarily at the prototypical phase. The significance of utilisation of combined drug therapies has been considered to be paramount from the perspective of application of clinical information collected from previous viral epidemics. One prospective treatment application has involved the Multi Drug Therapy (MDT) based approach with utilisation of the combination of drugs Nafamostat Mesylate and Favipiravir with the purpose of reduction of the infectious intensity of COVID-19 viral strain. On account of the extensive prevalence of patients becoming infected with the Novel Coronavirus strain, MDT procedures have been mostly favoured by scientists and clinical virologists with the explicit objective of determination of the probability of such combined drug therapies in terms of assisting the recovery of COVID-19 infected patients.

The previous researches conducted on the procedural particulars of treatments regarding effective antidote development for COVID-19 infected patients had brought forth various clinical outcomes on such innovative treatment initiatives concerning the observed effects of MDTs on such patients. The corresponding research literature review endeavour has been oriented towards collecting information regarding 2 specifically utilised medicinal substances (the previously mentioned Nafamostat Mesylate and Favipiravir drugs) for treatment purposes of COVID-19 infected individuals. Such drugs generally are associated with pharmaceutical categories such as antivirals, immune modulators, antibiotics and anticoagulants. These compounds have been utilised in a direct manner by hospital inpatients and such occurrences have permitted the researchers to examine the implications of such drugs on health conditions of COVID-19 patients in laboratory conditions as well. The corresponding study has been responsible for considering the clinical research findings of the combination of such drugs through comparison of observed outcomes.

## Highlights

• Novel type strain of coronavirus named SARS-CoV-2 is the causative agent of severe respiratory distress named COVID.
• Nafamostat Mesylate and Favipiravir reduce the infectious intensity of SARS-CoV-2 viral strain.
• Favipiravir strongly inhibits the enzyme RNA polymerase.
• Favipiravir was strongly considered to be as the choice for the inhibition of the SARS CoV-2 virus strain.
• Nafamostat Mesylate had anti-inflammatory and anti-coagulation effect in the severe conditions.

## 1 Introduction

It is also important to know about this virus as it has caused pandemic and changed the human history for the life time. This particular virus is a positive-sense single-stranded RNA virus of the size (26–32 kb) and it is from the family of the *Coronaviridae* in the Nidovirales of the genus named Betacoronavirus [1]

A novel type strain of coronavirus named SARS-CoV-2 is the causative agent for severe respiratory disease among human beings and the disease caused by it is named as Covid 19. The virus is easily transmittable from one human and eventually caused pandemic throughout the globe. The novel strain showed 80% homology with the type strain SARS-CoV which was responsible for acute respiratory distress syndrome (ARDS) that demonstrated high rate of mortality in the year, 2002–2003. As per statistical report of Centre for Systems Science and Engineering (CSSE) at John Hopkins University, nearly 1,400,000 cases of disease Coronavirus 19 have been reported till April 7th, 2020 [3].

## 2 Pathophysiology

SARS-CoV-2 especially have an effect on the respiratory system, in contrast to, other organs may also get affected and this cause multi-organ failure. The infection at lower respiratory tract causes symptoms such as fever, dry cough, dyspnea along with headache, dizziness, generalized weakness, vomiting and diarrhoea. Coronavirus is largely divided into following genera α, β, γ, and δ depending on their structural genome. The particular strains 229 E and NL63 belong to α group and causes common cold infections among humans. SARS-CoV-2 belongs to β virus**.** Corona virus is composed of 4 structural proteins: Spike (S), membrane (M), envelop (E) and nucleocapsid (N). The spike proteins of both SARS-CoV-2 and SARS-CoV attach to the **Angiotensin converting enzyme 2 (ACE2)**. ACE2 receptors are also expressed upon cells of organs lung, heart, ileum, kidney and bladder. This receptor is maximally expressed upon the lung epithelial cells through the virus enters and damages the cells. Epithelial cells, alveolar macrophages and dendritic cells (DCs) comprise the innate immunity in airway and T cell mediated response are also previously reported against coronavirus. Virus infected cells are phagocytosed by DCs which causes activation of CD4^+^ and CD8^+^ T cells. CD4^+^ T cells stimulate B cells to release virus specific antibody whereas CD8^+^ T cells kills virus infected cells. Severely infected patients were observed to demonstrate enhanced concentrations of proinflammatory cytokines within plasma, that includes interleukin (IL)-6, IL-10, granulocyte-colony stimulating factor (G-CSF), monocyte chemoattractant protein 1 (MCP1), macrophage inflammatory protein (MIP)1 α, and tumor necrosis factor (TNF)-α [4].

As per the observations of study by (Pal. Berhanu, Desalegn and Kandi, 2020) [5], specific therapeutic procedure development against the **Severe Acute Respiratory Syndrome Coronavirus 2 (**SARS-COV-2**)** is premised upon the measure of exigency which currently exists throughout the world in the form of the pandemic brought about by this particular viral pathogen. Multiple drugs, including the anti-malarial and anti-Ebola virus drugs have been brought under investigation to be applied against the pathological complications initiated by Coronavirus Disease 2019 (COVID-19). In this context, according to Rabaan et al. (2020) [6], the most significant contribution of entry of SARS-COV-2 into the cell cytoplasm of any individual, could be attributed to the enzyme Transmembrane pro-tease serine 2 (TMPRSS2). Thus, it has been argued by Mykytyn et al. (2021) [7], that inhibition of activity of TMPRSS2 is fundamental towards prohibition of the entry of SARS-COV-2 within bronchial cell cytoplasm. Thus, according to Mykytyn et al. (2021) [7], since the inception of the COVID-19 pandemic, there has been conducted the intensive screening of 1017 different, existing drugs. Out of such evaluated drugs, the **Nafamostat Mesylate**, a serine protease inhibitor which is clinically available, has been identified as the primary and most potent inhibitor for epithelial cell entry of Coronavirus which had been observed during the Middle East respiratory syndrome epidemic. To this effect, the research of (Yamamoto et al. 2016) have also highlighted that **Nafamostat Mesylate** has been proven to inhibit the SARS-COV-2 entry into human epithelial cells when EC_50_ of ~10 nM had been the clinical evaluation threshold [8]. Previously, **Nafamostat Mesylate** had been utilised for treatment of patients suffering from acute pancreatitis and in cases of intravascular coagulation where **Nafamostat Mesylate** had successfully disseminated such clots. Furthermore, at clinical level trials, as per the research of Ng et al. (2020), **Nafamostat Mesylate,** through intravenous administration within the blood circulation system, could sufficiently inhibit the viral entry of SARS-COV-2 (COVID-19) into human epithelial cell cytoplasm, however, the concentration of the drug within the blood of the patient would be required to be sustained at 30–240 nM [4].

On the other hand, according to Madappuram and Kamel (2020), Favipriavir has been the drug utilised with the purpose of administering treatment to H1NA virus previously [9]. This particular drug has exhibited effective antiviral capacity and activity against various RNA viruses [9]. To this effect, Favipriavir has been expected to exhibit effective antiviral qualities against the SARS-COV-2 as well [9]. Atzrodt et al. (2020) have also attested to the observation that inhibition of multiplicity of influenza viral strains had been performed by Favipriavir and such viral strains had been particularly resistant to differential drugs such as Rimantadine, Zanamivir, Amantadine and others [10]. Favipriavir functionalities involve an extensive of antiviral properties in cases of particularly drug resistant viral strains [10]. In case of the SARS-COV-2, the Favipriavir could effectively inhibit the virion M2 ion channel [10].

In this context, Bzikha, Bouhmou, Bzikha and Bouchnafati (2021), have outlined that the clinical study conducted at the Third People's Hospital within Shenzhen, PRC, had brought forth clinical outcomes of COVID-19 patients effectively administered and treated with Favipriavir and Atazanavir Versus Lopinavir/Ritonavir (LPV/RTV) [11]. The outcomes specified that patients administered with the Favipriavir drug demonstrated greater recovery rate [11]. Furthermore, the chest radiography of involved groups of patients demonstrated greater recovery transformations in case of patients administered with the Favipriavir in comparison to that of patients administered with LPV/RTV [11]. Furthermore, (Atzrodt et al. 2020) have emphasised on secondary studies conducted within the same hospital, involving clinical trial with patients infected by SARS-COV-2 virus and the clinical trial outcomes demonstrated comparatively greater antiviral response when such patients had been administered with Favipriavir [10]. However, particular side effects such as Nausea had been registered within patients who had been administered Favipriavir, thus, Ohe, Furuya and Goudarzi (2021) have argued in favour of the proposition that, in case of patients with previous history of immunological and respiratory complications, Favipriavir could be administered only in particular percentage-based combination with drugs which have had proven efficacy against SARS-COV-2 such as Nafamostat Mesylate [12].

From the research perspective of Asakura and Ogawa, (2020), this particular observation could be lent credence on account of the fact that Nafamostat Mesylate has been the primary medication utilised in treatment of DIC (Disseminated Intravascular Coagulation) as well as Pancreatitis previously [13]. In spite of the fact that Nafamostat Mesylate is primarily an anti-thrombin drug, this particular drug also exhibits the most powerful anti-plasmin characteristic [13]. Thus, this drug could be considered to be specifically suitable for complications which have enhanced fibrinolytic implications [13]. Furthermore, the Nafamostat Mesylate is particularly beneficial for COVID-19 patients since this drug does not contribute to haemorrhagic conditions internally [13]. Apart from these, according to (Asakura, and Ogawa, 2020), Nafamostat Mesylate could further block effectively the entry of viral strains into epithelial cells of humans through inhibition of membrane fusion between such cells and SARS-COV-2. Thus, efficacy of the drug towards impartation of effective treatment of COVID-19 patients has been the pertinent expectation on account of multiplicity of virologists and scientists [13].

## 3 Materials and methods

As per the research of Doi, Ikeda, Hayase, Moriya and Morimura (2020), the case study involving the treatment provided by the University of Tokyo Hospital between April 06, 2021 and April 21, 2021 to 11 adult patients, could be capitalised upon, from the clinical as well as academic perspective, in terms of evaluation of the efficacy of Multi Drug Therapy (MDT) based approaches in cases of treatment of patients suffering from SARS-COV-2 virus [14]. The adult patients had been admitted with SARS-COV-2 infection with reverse transcriptase polymerase chain reaction syndrome during the previously mentioned time duration [14]. They were admitted into the Intensive Care Unit (ICU) of the University of Tokyo Hospital and the treatment regime involved administering them with the combination of Nafamostat Mesylate and Favipiravir [14].

The clinical and demographic particulars and associated laboratory findings during the ICU admission had attested to the critical conditions during the initial phases of admission for such patients. Oxygen therapy had to be administered to every patient on account of their severe breathing complications. Furthermore, 8 out of 11 patients (73%) required Mechanical Ventilation (MV) of invasive manner. The remaining 3 patients (27%) required Venovenous extracorporeal membrane oxygenation [14].

Eight patients (73%) needed invasive mechanical ventilation (MV), and 3 patients (27%) needed vevovenous Patients received combination treatment with nafamo-stat mesylate [0.2 mg per kg per hour by continuous intravenous infusion, median treatment 14 days (IQR, 10–14 days)] and favipiravir [3600 mg on day 1 and at1600 mg per day on day 2 and subsequently mediantreatment 14 days (IQR, 12–14 days)]. No interruption of antiviral treatment occurred due to adverse drug reactions except for one patient who developed hyperkalemia on day 9 (by nafamostat mesylate). All 11 patients had at least 33 days of hospital follow-up. As of May 22, 1 patient, who had a do-not-resuscitate order, died on ICU day 7. Seven patients were successfully weaned from MV [median duration of MV 16 days (IQR, 10–19 days)] and 9 and 7 patients were discharged from the ICU and the hospital, respectively (Doi, Ikeda, Hayase, Moriya and Morimura, 2020) [14].

## 4 Theory

Multidrug therapy is a useful strategy to curb down the menace of many infectious diseases like leprosy, tuberculosis and to treat other fatal nosocomial infections. The basic mechanism behind the MDT treatment is that they simply slow down the rate of mutations or evolutions that leads to the development of the resistant bacteria or viruses etc [4]. The therapy based on polydrug treatment can be applied based on the two ways: one is at the hospital level and other is at the individual patient level. In this respect, it must be taken into notice that our World Health Organization have recommended that MDT can be applicable to the fatal patients of leprosy, tuberculosis at free of any treatment expenditure as these two diseases had already turned into an epidemic one. Because of the rapid pandemic spread of the Covid 19 viral pneumonia which is also associated with unparallel level of morbidity of patients, there is surely an urgent surge for the development of combinatorial drugs to curb down the growing menace and the suffering of the people due to the rising cases of deaths day by day [12]. In this moment of crisis, the technique called the drug repurposing, i.e., that is depicting the commercially available patented medications as an alternative would rapidly minimize the expenditure and would result in something feasible other than explorations of something entirely new path or “***de novo* treatment strategy**”. In the very recent times many drugs have gone into testing phase to cure down the patients of Covid 19 such as the drugs like Hydroxychloroquine, Favipiravir, Azithromycin, Remdesivir, Nafamostat mesylate, and Lopinavir/Ritonavir, and many such medications. Though such medications have been recently tried against the fatal pandemic disease, there is hardly any successful result alone.

## 5 Results

**Fig. 1:**
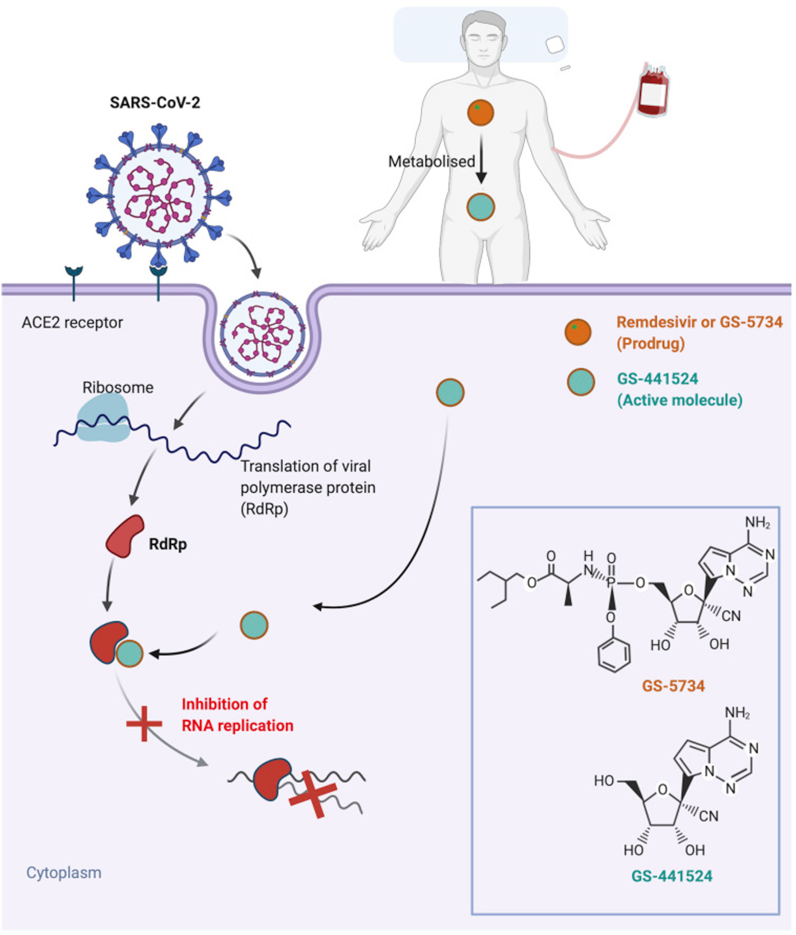
Mechanism of action explanation of drug Remdesivir against Covid 19.

Source: (Andri Frediansyah, Firzan Nainu, Kuldeep Dhama, Mudatsir Mudatsir, Harapan Harapan, 2020) [2].

Remdesivir and Favipiravir are extremely important group of drugs, though it has certain side effects, though one thing that its proficiency has still not been approved after several repeated trials. We can refer to one of the author's mechanism of action explanation regarding this matter (see Fig. 1).

Now, it must be mentioned that with Favipiravir, as the candidate medications many specialized trials are on its way and also observational investigation is also going to progress as the sponsor-commenced clinical investigation. The mechanism of action of the above mentioned medication is that it strongly inhibits the enzyme RNA polymerase and therefore it was strongly considered to be as the choice for the inhibition of the SARS CoV 2 virus strain (see Fig. 1). The other drug namely Nafamostat mesylate is basically an anticoagulant agent and also it has its application already in the country Japan for the treatment of the acute intra-vascularitis and pancreatitis. Therefore, the combinatorial therapy of both of the potential medications might help in the treatment of the fatal pandemic disease. The mechanism of action may be action on the varied parts of the replication or the multiplication of the virus unit, and therefore again it is expected to have an atleast additive effect when the two medications will be used together. As most of the Covid 19 patients suffers from the coagulation of the blood intravascular conditions and it can be pathologically fatal, so in that condition the drug Nafamostat mesylate will take care of the fatal condition [15].

## 6 Discussion

In that case if this particular combinatorial therapy proved to be an efficacious one then one might be able to formulate an effective antiviral therapy to successfully treat Covid 19 patients but the drugs should not contra-interact. The drug, Nafamostat mesylate also has been observed that it strongly inhibits the entry of the virus within the cells of the human. This experiment was carried out at the Institute of Medical Science of The University of Tokyo (IMSUT). Favipiravir was also approved by the Ministry of Health, Labour and Welfare at the date of March 2014, however, it was observed that it has limited efficiency in the treatment the novel pandemic influenza. Behind this the drug has the mechanism of action that it converts the ribosyl triphosphate within the physiological system and also depicts particular interference to the RNA polymerase enzyme of the virus. It also shows that this particular drug also shows action against different RNA virus other than the influenza virus. Many of the research scholars or the scientists are working with this particular drug against many viruses or against this virus within varied clinical trials [15]. The drug Nafamostat mesylate reveals inhibitory action against the enzymes that degrades proteins, so this particular medication is used for ameliorating the condition of the patient suffering from the acute pancreatitis. This particular drug has been used for many of the years in the country Japan. It was the Jun-ichiro Inoue group working at the Institute, IMSUT highlighted that this particular medication also have the capacity to inhibit at the action mechanism of SARS CoV −2. Those viral infections that involve the infection of the respiratory tract, the EC50 value (the value or the concentration or the dosage of the drug where it inhibits the infection of the virus by about 50%) of the medication named Nafamostat mesylate showed to be 10 nM only which is itself a extremely low concentration [14]. Moreover, many paients have reported the pathological condition of rapid blood clot within the blood arteries in the severe conditions only and in that aspect also Nafamostat mesylate is also considered to be an ideal consideration for the patients. So apart from the antiviral activity this activity is also considered to be a significant action. This has been observed within a clinical study that is single blinded randomized controlled, comparative investigation and which is also a multicentre one.

The outbreaks of this pandemic infection by this virus have already resulted in an unparallel economic and social devastation. The unique feature of this pandemic is that it can depict symptomatic manifestation from normal respiratory syndrome to acute respiratory syndrome (ARDS). It has been also observed that corona virus during the past 3 years have shown lethal infections. Moreover, the rapid spread of this disease has called for an urgency to conduct world-wide research investigation [14].

## 7 Conclusion

This particular virus is a positive-sense single-stranded RNA virus of the size (26–32 kb) and it is from the family of the *Coronaviridae* in the Nidovirales of the genus named *Betacoronavirus*. A novel type strain of coronavirus named SARS-CoV-2 is the causative agent for severe respiratory disease among human beings and the disease caused by it is named as Covid 19. The virus is easily transmittable from one humans and eventually caused pandemic throughout the globe.

Alleviation and treatment of the extensive detrimental implications of COVID-19 have materialised into the primary objectives of scientists and virologists along with pathologists assigned with the responsibility of provisioning of care for infected patients. Development and introduction of vaccines have been, till to date, primarily at the prototypical phase. Now, it must be mentioned that with Favipiravir, as the candidate medications many specialized trials are on its way and also observational investigation is also going to progress as the sponsor-commenced clinical investigation. The mechanism of action of the above mentioned medication is that it strongly inhibits the enzyme RNA polymerase and therefore it was strongly considered to be as the choice for the inhibition of the SARS CoV 2 virus strain. The drug Nafamostat mesylate reveals inhibitory action against the enzymes that degrades proteins, so this particular medication is used for ameliorating the condition of the patient suffering from the acute pancreatitis. This particular drug has been used for many of the years in the country Japan. It was the Jun-ichiro Inoue group working at the Institute, IMSUT highlighted that this particular medication also have the capacity to inhibit at the action mechanism of SARS CoV −2. Those viral infections that involve the infection of the respiratory tract, the EC50 value (the value or the concentration or the dosage of the drug where it inhibits the infection of the virus by about 50%) of the medication named Nafamostat mesylate showed to be 10 nM only which is itself a extremely low concentration. Moreover, many paients have reported the pathological condition of rapid blood clot within the blood arteries in the severe conditions only and in that aspect also Nafamostat mesylate is also considered to be an ideal consideration for the patients. The particular strains 229 E and NL63 belong to α group and causes common cold infections among humans. SARS-CoV-2 belongs to β virus**.** Corona virus is composed of 4 structural proteins: Spike (S), membrane (M), envelop (E) and nucleocapsid (N). The spike proteins of both SARS-CoV-2 and SARS-CoV attach to the Angiotensin converting enzyme 2 (ACE2). Thus in this write up we could show the multidrug therapy of the two above said medications and their working mechanism against the Covid 19 affected serious patients.

## Provenance and peer review

Not commissioned, externally peer-reviewed.

## Ethical Approval

No Please state whether Ethical Approval requested.

## Sources of funding

No any sources of funding for your research.

## Author contribution

Main Author and single Author Maram Hussen Abduljabbar.

## Research registration Unique Identifying number (UIN)

Name of the registry:

Unique Identifying number or registration ID:

Hyperlink to your specific registration (must be publicly accessible and will be checked):

## Guarantor

Maram Hussen Abduljabbar.

## Declaration of competing interest

No any conflicts of interest.
